# How artificial intelligence will affect the future of medical publishing

**DOI:** 10.1186/s13054-023-04511-9

**Published:** 2023-07-06

**Authors:** Jean-Louis Vincent

**Affiliations:** Department of Intensive Care, Erasme Hospital, Université Libre de Bruxelles, Route de Lennik, 1070 Brussels, Belgium

There is no question that artificial intelligence (AI) will (is already) radically transform(ing) the world of medical publishing, for the better, much more than for the worse. For researchers and journal editors, AI-based systems will enable more complex problems to be addressed, based on input from multiple sources of information, an approach that is not possible or would be impossibly slow without assistance from AI.

The benefits of AI in medical publishing can be considered in terms of their effects on three aspects: content, peer review, and post-publication (Fig. 1). AI will speed up each of these processes and make them more accurate and efficient.

**Fig. 1:**
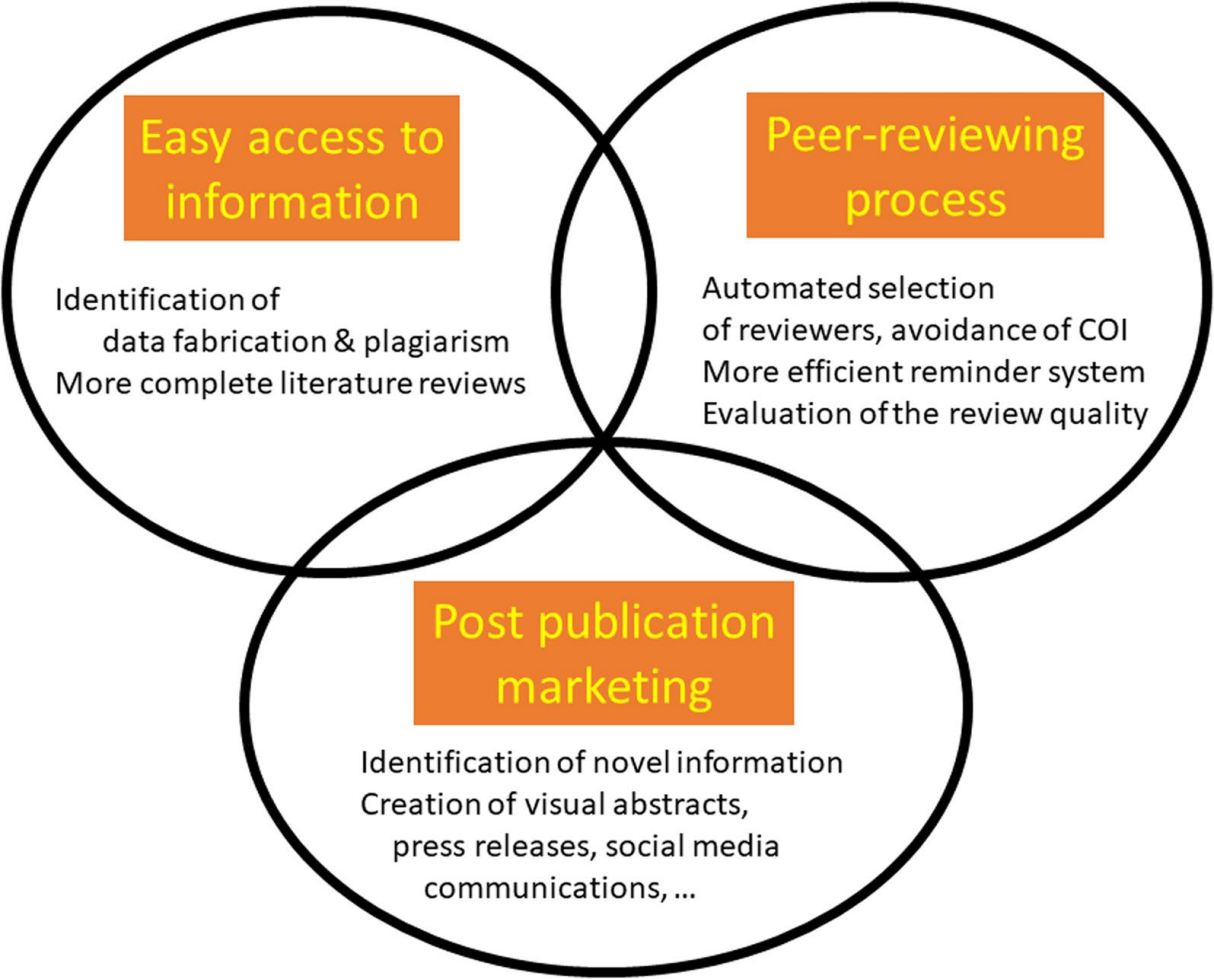
Three key aspects of medical publishing that can be improved using artificial intelligence (AI)

Most research starts with a comprehensive synthesis of current knowledge on a particular question—the literature review. There have been three key periods in this process over the years. The first period (from 1879 to 2004) was dominated by Index Medicus and essentially involved having to go to the library to look for a precise reference in the large Index books, finding the relevant issue of the journal (if stocked) on the library shelf, and then making a ‘xerox copy.’ This process needed to be repeated for each article! The second period is the current one, with indexing now online, largely via PubMed. This database provides a selection of references based on keywords introduced into the search engine. Many of the journal articles retrieved in such a search are now open access (including those published in *Critical Care*, of course) and can be downloaded to one’s PC or reference manager; printing has largely been abandoned. Although much simpler and faster than in the past, the literature review process using PubMed is still time consuming and influenced by a researcher’s preferences and biases. In the third period, which we are now entering, literature reviews will be even easier, yet much more complete and also more objective, retrieving a broader, more extensive collection of references. AI-based systems will not only access more articles, but they will also automatically select the most relevant and analyze their quality. As Salvagno and Taccone recently underlined [1], the amount of information now available has become so vast that it is impossible for us to take it all into account and integrate it into any research project. AI can help not only to identify all the relevant information, but also to organize it in a meaningful way.

For researchers, AI-based systems will thus be of tremendous help. As discussed, they can already help collect, collate and classify huge amounts of relevant data on a chosen subject or research question—much better than a PubMed search! AI can also help to generate a hypothesis, select the most appropriate end-points, and improve a study protocol, including the statistical analysis [2].

AI-based systems will also help researchers improve the quality of their writing. Although there is some concern that systems like ChatGPT will be used to actually write texts, I would argue that this is not a problem. On the contrary, AI assistance is welcome if it improves the way in which the data are interpreted and integrated into an article. Too many papers are poorly structured, include repetitions and redundant text, and need language editing due to poor command of the English language. The clarity and quality of papers could be much improved by using AI.

For journal editors, AI systems can help check the quality and completeness of studies in submitted articles. Even well-known and frequently cited studies published in major journals can have important shortcomings. For example, a famous trial, the results of which were published in the JAMA [3], compared infusions of sodium chloride and a balanced solution, but measurements of blood chloride levels, which were essential to interpretation of the results, had been forgotten [4].

The risks of data fabrication and plagiarism are serious issues, but here also AI will be of help to identify and prevent such events (using AI systems to recognize AI-generated data). Data fabrication will always lead to some incongruity and inconsistency. In the medical field, it was the implausibility of the standard deviations of some variables that eventually led to the recognition of scientific misconduct by Joachim Boldt, with the retraction of the vast majority of his numerous publications [5]. As AI systems are used more widely, the large amounts of input data will result in easier identification of data fabrication and detection of plagiarism.

AI-based systems will also help editors in their task to find the most appropriate reviewers for specific papers, based on reviewers’ backgrounds, lists of publications (including those cited in the paper to be reviewed), and previous reviewing records (how well and how fast they reviewed previous papers). The system could even take care of the whole process, from selection of experts, through invitations and collection of reports, to a final decision based on the reviewers’ recommendations. In a provocative letter, Salvagno and Taccone [1] propose that AI systems could replace the Editor-in-Chief, providing a more objective and more efficient process than is currently the case. Post-publication queries about integrity, errata, and retractions could also be reduced by using a complete AI-based editorial process.

The post-publication impact of articles and spread of information will also be facilitated, by automatic identification of the important novel aspects of published research, leading to more rapid application of effective new concepts and/or meaningful improvements in practice. Press releases, visual abstracts, summary videos, and social media posts can all be created by AI, and targeted appropriately at specific populations, from the specialist through to the general public.

The increasing role of AI technology in the future is evident and should be seen as a positive event as it will be of tremendous help to assist both authors and publishers. As an Editor-in-Chief, I do not feel threatened, but relieved that some of the more time-consuming and complex aspects of my job will be taken over, significantly improving the ultimate quality and output of the journal, which can only be for the good of science.

## Data Availability

Not applicable.
